# Resilience and empathy in pharmacy interns: Insights from a three-year cohort study

**DOI:** 10.1016/j.rcsop.2023.100333

**Published:** 2023-09-24

**Authors:** Syafiqah Nadiah Halimi, Ardalan Mirzaei, Debra Rowett, Karen Whitfield, Karen Luetsch

**Affiliations:** a School of Pharmacy, The University of Queensland, 20 Cornwall Street, Woolloongabba, Queensland 4102, Australia; b Kulliyyah of Pharmacy, International Islamic University Malaysia, 25710 Kuantan, Pahang Darul Makmur, Malaysia; c School of Pharmacy, Faculty of Medicine and Health, The University of Sydney, New South Wales 2006, Australia; d School of Pharmacy and Medical Sciences, University of South Australia, Adelaide, South Australia 5000, Australia

**Keywords:** Resilience, Empathy, Pharmacy interns, Surveys, Quantitative, Qualitative, Content analysis, COVID-19 pandemic

## Abstract

**Background:**

Resilience and empathy are important attributes for healthcare professionals to navigate challenging work environments and providing patient-centred care. Knowledge about pharmacists' levels of resilience and empathy, particularly during the early stages of their careers, is limited.

**Objectives:**

To explore pharmacy interns' levels of resilience and empathy using the Connor-Davidson-Resilience-Scale-25 (CD-RISC-25) and the Kiersma-Chen-Empathy-Scale (KCES), examine potential associations with demographic characteristics and ascertain what challenges interns' resilience and which support mechanisms they identify.

**Methods:**

Hard copies of the surveys were distributed to three cohorts during face-to-face intern pharmacy workshops from 2020 to 2022. Additionally, a qualitative questionnaire explored interns' experiences while completing an accredited internship program during the COVID-19 pandemic. Data were analysed using descriptive and inferential statistics, open-ended questions were analysed through qualitative and quantitative content analysis.

**Results:**

Among 134 completed surveys, most respondents were female, aged 18–22, and worked in hospitals. The CD-RISC-25 mean score was 66.6 (SD 11.7) and the KCES mean was 84.3 (SD 9.23) indicative of intermediate levels of resilience and empathy. Resilience and empathy scores did not significantly differ between the three cohorts (*p-value* > 0.05), and both were not consistently correlated with each other (*p-value* > 0.05). No significant associations were found between demographic characteristics and resilience scores. However, age and pre-internship employment history showed a statistically significant association with empathy scores (*p-value* < 0.05), with younger age groups and those who worked part-time during undergraduate studies demonstrating higher levels of empathy. Challenges undermining interns' resilience included the COVID-19 pandemic, internship requirements, and feelings of inadequacy and inexperience.

**Conclusions:**

This study showed that resilience and empathy scores among interns were at what can be regarded as intermediate levels, largely unaffected by the COVID-19 pandemic or cohort demographics. It highlights professional aspects and strategies which are professionally sustaining and may assist interns in navigating challenges to their resilience and empathy.

## 1 Introduction

Resilience reduces the risk of burnout for healthcare professionals and contributes to their long-term career success and satisfaction in complex and demanding practice environments.^1^ Resilience is broadly defined as the dynamic process of adapting to and recovering from challenges and dealing constructively with adversarial experiences, such as, difficult work situations, environments and crises.^2^^,^^3^

Burnout is regarded as a leading cause contributing to healthcare professionals leaving their profession,^4^ with several studies suggesting an inverse relationship between resilience and burnout.^5^ Like other healthcare professionals, pharmacists experience burnout and stress.^6^ Rates of burnout vary significantly between studies and surveys. For example, in 2017, over 61% of hospital clinical pharmacists reported burnout in the USA,^7^ whereas an earlier survey found one in twenty Australian hospital pharmacists experienced burnout.^8^ Early career healthcare professionals are at particular risk of burnout, for example, pharmacy residents perceive higher stress levels than those reported by adults aged 18 to 29.^8^, ^9^, ^10^ Australian pharmacists and interns under the age of 30 were found to be most vulnerable, regardless of their work setting.^11^

The COVID-19 pandemic added pressure to the roles of pharmacists, who were at the frontline of assuring and educating patients, ensuring medication supply, providing telehealth consultations, establishing vaccination centres, contributing to timely research and data analysis, and often working with limited personal protective equipment and workforce shortages.^12^^,^^13^ Australian pharmacists reported higher burnout scores during the COVID-19 pandemic than before it started.^8^^,^^14^

Enhancing resilience of pharmacists at the start of their professional careers may provide them with personal resources to recognise and respond constructively to current and future work-related pressures, reduce their likelihood of experiencing burnout and leaving the workforce prematurely.^15^

Another attribute sought after in healthcare professionals is empathy, which allows them to build relationships with patients but also conveys the ability to take care of themselves.^16^ Empathy is regarded as a core competency across medical and health professional training and is defined as “the act of correctly acknowledging the emotional state of another without experiencing that state oneself”.^17^ Healthcare professionals showing empathy improve patients' health outcomes and empathy underpins patient-centred care.^18^^,^^19^ Empathy and resilience may be positively linked; for example, physicians reported increased empathy after receiving resilience training.^20^ Similarly to resilience, empathy was found to be inversely correlated to burnout.^21^

Despite the wealth of literature on the importance of resilience and empathy among healthcare professionals and students, few studies have explicitly examined how resilient or empathetic pharmacists are at the start of their professional career.^11^^,^^22^^,^^23^ Pharmacy interns face many challenges that may undermine their resilience and empathy which include balancing academic work and intern examinations with their newfound professional responsibilities and demanding workloads. The COVID-19 pandemic has exacerbated these challenges by reducing supervision and preceptor contact.^24^^,^^25^ Knowing more about pharmacy interns' levels of resilience and empathy will inform considerations about instituting strategies which may strengthen both attributes with their potential to reduce burnout for this group of young healthcare professionals.

To date, there is no universally accepted benchmark for measuring resilience and empathy levels, but a number of instruments have been developed and validated.^26^^,^^27^ For example, the Connor-Davidson-Resilience-Scale 25 (CD-RISC-25) is a commonly used instrument to measure resilience. It has been translated into many languages, and its psychometric reliability, validity and population means have been established.^28^, ^29^, ^30^

Empathy in healthcare professionals is often measured with the Jefferson Scale of Empathy (JSE),^31^ and the Kiersma-Chen-Empathy Scale (KCES) was specifically designed to measure both the cognitive and affective domains of empathy in pharmacy and nursing students and professionals. It is closely modelled on the widely used JSE and reliability analysis confirmed that KCES ratings were favourably correlated with JSE ratings (*p* < 0.001).^32^

The aim of this study was to examine resilience and empathy in pharmacy interns using two validated psychometric instruments; the CD-RISC -25 and the KCES, to explore potential associations and relationships between resilience and empathy levels and demographic characteristics. This study also explored pharmacist interns' experiences which may affect their resilience and empathy while completing an accredited pharmacy internship program at an Australian university that coincided with the COVID-19 pandemic.

## 2 Methods

A cross-sectional survey was conducted among pharmacy interns enrolled in an Australian accredited internship training program (ITP) over three consecutive years (2020−2022). Ethical approval was obtained from the University of Queensland (HREC reference number: 2020000961). Participants provided written consent to participate. The Consensus-Based Checklist for Reporting of Survey Studies (CROSS) Guidelines was followed in conducting and reporting this study (Supplementary file 1).^33^

The survey consisted of three sections: demographic data collection, validated psychometric questionnaires and open-ended questions. Demographic characteristics collected were: age, gender, cultural background, education, previous and/or current employment status, and setting of internships (hospital, community). Psychometric questionnaires used were the CD-RISC-25 and KCES. Five open-ended questions adopted from an American pharmacist resilience and well-being survey asked interns what challenged and supported them in their professional work.^34^ The combined survey was anticipated to take 15 to 20 min to complete.

### 2.1 Connor-Davidson Resilience Scale-25 (CD-RISC-25)

The 25-item scale assesses five interrelated parameters: “personal competence”, “trust/tolerance/strengthening effects of stress”, “acceptance of change and secure relationships”, “control”, and “spiritual influences”. Responses are given on a five-point Likert scale ranging from 0 (completely disagree) to 4 (completely agree). The overall score is calculated by adding responses from all 25 items, with higher scores indicating greater resilience. The total possible scores range from 0 to 100.^35^

### 2.2 Kiersma-Chen-Empathy Scale (KCES)

The 15-item questionnaire assesses two types of empathy: “affective empathy” and “cognitive empathy”, with higher scores indicating greater empathy (range 15–105). Each item is rated on an ordinal scale from 1 to 7, indicating how much the item matches participants' perceptions and feelings about a particular patient group or action. Four of the items are negatively worded and coded in reverse order.^32^

### 2.3 Open-ended questions

Open-ended questions asked pharmacy interns to describe personal experiences which support their professional satisfaction and fulfilment, their needs in sustaining them, as well as their sources of professional stress. Questions and the coding scheme for answers were adopted from Schommer and colleagues.^34^

Pharmacy interns in this study were graduates who had completed a four-year bachelor's degree and were undertaking a period of supervised practice following the requirements set out by the Pharmacy Board of Australia.^36^ Utilising convenience sampling, all interns attending an ITP workshop in person while enrolled in a university-based, accredited ITP in Australia from 2020 to 2022 were invited to fill out a paper-based survey. The workshop attendance rate was 90% (72 out of 80) in 2020 and 100% (69 out of 69) in 2022. In 2021, 60.3% (35 out of 58) of interns were able to attend the workshop in person, due to travel restrictions during the COVID-19 pandemic.

## 3 Data collection and analysis

Surveys were distributed manually during the ITP workshop every September from 2020 to 2022, towards the end of their internship program. Participation was voluntary, and no incentives were provided to participants.

This study used quantitative and qualitative analytical methods. Descriptive statistics summarise participants' demographic data. Data verification was performed before analysis, and only fully completed CD-RISC-25 and KCES responses were included. Missing data from incomplete surveys were handled through listwise deletion.^37^

The D'Agostino-Pearson normality test was used to determine the distribution of data. The CD-RISC-25 resilience data were normally distributed (*p* > 0.05); therefore, parametric tests (independent *t*-test and one-way ANOVA) were used to investigate factors associated with resilience scores. Empathy data were not normally distributed (*p* < 0.05), and non-parametric tests, such as Mann-Whitney and Kruskal-Wallis tests, were performed to determine if significant differences existed between demographic characteristics and empathy levels. The Spearman rank correlation was used to analyse relationships between the two research variables: resilience and empathy.

All data were entered into CheckBox® (Checkbox Survey, Inc., MA, USA), and then imported into Microsoft Excel® before being analysed using GraphPad Prism version 9® (GraphPad Software, California USA). A threshold of *p* < 0.05 was used to determine statistical significance.

The open-ended questions in Section 3 were analysed using qualitative and quantitative content analysis. The qualitative approach focused on interpreting themes and patterns, through a deductive process which started with categorising data under codes and themes developed and presented in the original article by Schommer et al. (2020),^34^ with development of additional themes for data which could not be categorised in this manner, for example, those relating to the COVID-19 pandemic.^38^ Quantitative methods were utilised to quantify interns' responses, specifically through the counting of particular words or phrases which exemplified the codes and themes, providing a measurable insight into the frequency of certain themes.^39^

## 4 Results

A total of 139 pharmacy interns filled out the surveys from 2020 to 2022, with 134 included in the analysis. Five (<4%) responses were missing data and were not included in the analysis, and the Grubbs' test returned no outliers. The group consisted of 99 females (73.9%) and 35 males (26.1%).

The total number of pharmacy interns enrolled in the ITP program from 2020 to 2022 was 207. Of these, 176 were able to attend the in-person workshops. The response rates for each year are based on attendee numbers as only these were able to fill out the survey and are as follows: 77.8% (56 out of 72) in 2020; 48.6% (17 out of 35) in 2021; and 88.4% (61 out of 69) in 2022. The majority of respondents were working in a hospital (68.8%), 18–22 years of age (47%) and held a bachelor's degree (94.8%). The demographic characteristics of the pharmacy interns are shown in Table 1.

**Table 1:** Demographic characteristics.

Variable	Characteristics	2020	2021	2022	Overall
		n (%)			
Total		56 (100)	17 (100)	61 (100)	134 (100)
Age	18–22 years	22 (39.3)	7 (41.2)	34 (55.7)	63 (47.0)
	23–27 years	21 (37.5)	7 (41.2)	21 (34.4)	49 (36.6)
	>28 years old	13 (23.2)	3 (17.6)	6 (9.8)	22 (16.4)
Gender	Female	37 (66.1)	13 (76.5)	49 (80.3)	99 (73.9)
	Male	19 (33.9)	4 (23.5)	12 (19.7)	35 (26.1)
Internship Settings	Community pharmacy	15 (26.8)	3 (17.6)	21 (34.4)	39 (29.1)
	Hospital pharmacy	38 (67.9)	14 (82.3)	40 (65.6)	92 (68.6)
	Others	3 (5.3)	0 (0)	0 (0)	3 (2.3)
Previous Education	Bachelor's degree	51 (91.1)	16 (94.1)	60 (98.4)	127 (94.8)
	Master's degree	3 (5.4)	1 (5.9)	1 (1.6)	5 (3.8)
	Postgraduate diploma	1 (1.8)	0 (0)	0 (0)	1 (0.7)
	Vocational training	1 (1.8)	0 (0)	0 (0)	1 (0.7)
Previous (pre-intern) Employment History	Employed full-time	12 (21.4)	5 (29.4)	14 (23.0)	31 (23.1)
	Employed part-time	42 (75)	12 (70.6)	46 (75.4)	100 (74.6)
	No working history	2 (3.6)	0 (0)	1 (1.6)	3 (2.3)
Duration (in months)	Mean (SD)	40.1 ± 30.8	47.1 ± 56.6	33.1 ± 20.4	38.7 ±33.8
Current Employment (while doing internship)	Employed full-time	55 (98.2)	17 (100)	59 (96.7)	131 (97.7)
	Employed part-time	1 (1.8)	0 (0)	2 (3.3)	3 (2.3)
Duration (in hours)	Mean (SD)	39.1 ± 3.06	39.7 ± 0.73	39.8 ±4.30	39.4 ±3.41

### 4.1 Connor-Davidson Resilience Scale-25 (CD-RISC-25)

The reliability of the CD-RISC-25 was assessed using Cronbach's alpha coefficient, which was 0.81, indicating good internal consistency. The pharmacy interns' mean score, standard deviation, and median scores for the CD-RISC-25 are provided in Table 2. The mean CD-RISC resilience score was 66.6 ± 11.7, with total scores ranging from 36 to 100. As recommended by the CD-RISC-25 manual resilience scores were stratified into quartiles (Table 2) with the median score as the midpoint of the score distribution. The first quartile represents low resilience, the second and third quartiles represent intermediate resilience, and the fourth quartile represents high resilience.^35^

**Table 2:** Analysis of CD-RISC-25 scores and CD-RISC-25 quartiles.

CD-RISC-25 scores												
Year	2020			2021			2022			Overall		
	Total	Female	Male	Total	Female	Male	Total	Female	Male	Total	Female	Male
N cases	56 (100)	37 (66.1)	19 (33.9)	17 (100)	13 (76.5)	4 (23.5)	61 (100)	49 (80.3)	12 (19.7)	134 (100)	99 (73.9)	35 (26.1)
Mean (SD)	65.9 ± 12.0	65.2 ± 11.6	67.3 ± 13.1	70.2 ± 12.3	71.2 ± 12.7	67.3 ± 12.4	66.2 ± 11.2	66.4 ± 10.8	65.6 ± 13.4	66.6 ± 11.7	66.6 ± 11.4	66.7 ± 12.8
Median (Min-Max)	67 (39–100)	67 (44–100)	68 (39–85)	70 (49–96)	68 (55- 96)	72 (49–76)	66 (36–93)	67 (36- 93)	63 (45–91)	67 (36–100)	67 (36–100)	68 (39–91)

CD-RISC-25 quartiles												
Quartiles/Year	2020			2021			2022			Overall		
	n (%)											
First quartile	14 (25)			4 (23.5)			15 (24.6)			29 (21.6)		
Second quartile	12 (21.4)			4 (23.5)			13 (21.3)			35 (26.1)		
Third quartile	16 (28.6)			5 (29.5)			18 (29.5)			34 (25.4)		
Fourth quartile	14 (25)			4 (23.5)			15 (24.6)			36 (26.9)		

### 4.2 Kiersma-Chen-Empathy Scale (KCES)

The Cronbach's alpha value for the scale was 0.75, which suggests an acceptable range of internal consistency among the items. The pharmacy interns' mean score, standard deviation, and median scores for the KCES are provided in Table 3. The total mean KCES score for pharmacy interns was 84.3 ± 9.23, with total scores ranging from 55 to 98. Empathy levels were stratified by quartiles with the median score representing the midpoint of the frequency distribution (Table 3).

**Table 3:** Analysis of KCES scores and KCES quartiles.

KCES scores												
Year	2020			2021			2022			Overall		
	Total	Female	Male	Total	Female	Male	Total	Female	Male	Total	Female	Male
N cases	56 (100)	37 (66.1)	19 (33.9)	17 (100)	13 (76.5)	4 (23.5)	61 (100)	49 (80.3)	12 (19.7)	134 (100)	99 (73.9)	35 (26.1)
Mean (SD)	83.6 ± 12.0	85.0 ± 9.12	80.9 ± 11.5	86.8 ± 6.08	85.8 ± 6.59	89.8 ± 2.75	84.3 ± 9.16	85.4 ± 8.7	79.8 ± 9.96	84.3 ± 9.23	85.3 ± 8.55	81.5 ± 10.6
Median (Min-Max)	85 (55–97)	85 (55- 97)	79 (62–96)	87 (71–97)	86 (71- 97)	89.5 (87–93)	85 (60–98)	86 (61- 98)	80 (60–96)	85.5 (55–98)	86 (55- 98)	81 (60–96)

KCES quartiles												
Quartiles/Year	2020			2021			2022			Overall		
	n (%)											
First quartile	14 (25.0)			4 (23.5)			14 (23.0)			30 (22.4)		
Second quartile	13 (23.2)			3 (17.6)			15 (24.6)			37 (27.6)		
Third quartile	15 (26.8)			6 (35.4)			16 (26.2)			33 (24.6)		
Fourth quartile	14 (25.0)			4 (23.5)			16 (26.2)			34 (25.4)		

Resilience and empathy scores showed no significant differences between different ITP cohorts (2020–2022). No significant differences between demographic characteristics (age, gender, internship settings, cultural background, previous education and employment history) and the overall CD-RISC-25 score was found on one-way ANOVA and independent t-test. Supplementary file 2 shows the association of demographic characteristics with overall CD-RISC-25 and KCES scores.

The effect of demographics on the KCES scores was examined using the Kruskal-Wallis and Mann-Whitney tests. The results of the Kruskal-Wallis test showed a statistically significant effect of age (H(2) = 7.90, *p-value* < 0.05), indicating that at least one age group had significantly different median scores compared to the others. Follow-up analysis with the Mann-Whitney U test indicated that younger age groups, 18–22 (p-value < 0.01) and 23–27 years old (p-value < 0.05) scored significantly higher than the older age group (>28 years old). The median scores were 86 for both younger groups and 81 for those above 28 years.

There was a statistically significant difference between the median KCES scores and pre-intern employment histories, as shown by the Kruskal-Wallis test (H(2) = 9.78, p-value < 0.05)*.* No other demographic characteristics (gender, internship settings, cultural background and previous education) influenced KCES scores.

Spearman rank correlation was used to examine the relationship between CD-RISC-25 and KCES scores, *p-value* > 0.05 (not significant), 95% confidence interval − 0.117 to 0.231, *r* = 0.06. This showed that resilience and empathy scores in this cohort are not consistently positively or negatively correlated. Nevertheless, 10% of participants scored in the lowest quartiles for both empathy and resilience, whereas 8.9% were in the highest quartile for both.

### 4.3 Open-ended responses

The frequency of answers to the five open-ended questions, which were categorised using the coding scheme and themes developed by Schommer et al. (2020) are shown in Table 4.^34^ The findings from pharmacy interns are mostly similar to the original survey of pharmacists and pharmacist students with some differences related to the study focusing on interns and being conducted during the COVID-19 pandemic, resulting in additional themes and prioritisations.

**Table 4:** Pharmacy interns' themed responses.

No	Question	Theme	2020	2021	2022
			n (%)		
1.	What are the positive things in your professional life that give you satisfaction and fulfilment?	Helping patients	31 (39.2)	11 (42.4)	43 (52.5)
		Professional development	22 (27.8)	7 (26.9)	21 (25.7)
		Relationship with co-workers/colleagues	18 (22.9)	3 (11.5)	8 (9.6)
		Mentoring/feedback	3 (3.8)	3 (11.5)	5 (6.1)
		Making a difference	5 (6.3)	2 (7.7)	5 (6.1)
2.	What are the things that you view as specific stressors in your professional life?	Feeling inadequate and inexperienced	21 (26.4)	4 (13.8)	19 (23.5)
		Inadequate recognition	5 (6.2)	9 (31.0)	10 (12.3)
		Time management	7 (8.7)	6 (17.2)	18 (22.2)
		Organisational management	7 (8.7)	6 (20.7)	15 (18.5)
		ITP requirements	9 (11.3)	4 (13.8)	9 (11.1)
		Angry/abusive patients & customers	14 (17.5)	0	5(6.2)
		COVID-19 pandemic	12 (15.0)	0	2 (2.5)
		Finances	5 (6.2)	0	3 (3.7)
3.	What interferes with your ability to meet your expectations in your professional life?	Time management	8 (14.2)	7 (29.2)	26 (34.7)
		Organisational management	12 (21.4)	5 (20.8)	15 (20.0)
		Health	17 (30.4)	3 (12.5)	20 (26.7)
		Striving for perfection	11 (19.7)	6 (25.0)	9 (12.0)
		Relationships	6 (10.7)	3 (12.5)	3 (4.0)
		Finances	2 (3.6)	0	2 (2.6)
4.	What needs do you have in your professional life that would enable you to achieve satisfaction and fulfilment?	Organisational support	11 (23.4)	6 (27.3)	17 (25.4)
		Professional development	12 (25.6)	6 (27.3)	15 (22.4)
		Acknowledgement and recognition	8 (17.0)	2 (9.0)	10 (14.9)
		Time management	7 (14.9)	6 (9.0)	7 (10.5)
		Relationships	4(8.5)	2 (9.0)	10 (14.9)
		Health	5 (10.6)	0	8 (11.9) 22.5
5.	What are specific actions in your professional life that you could take to allow you to achieve satisfaction and fulfilment?	Professional development	27 (65.8)	8 (38.1)	26 (51.0)
		Time management	7 (17.1)	8 (38.1)	14 (27.5)
		Mindfulness/reflection	2 (4.9)	4 (19.0)	9 (17.6)
		Asking for help	5 (12.2)	1 (4.8)	2 (3.9)

In answer to the first question, pharmacy interns expressed that helping patients and professional development are among the positive aspects of their professional practice, giving them satisfaction and fulfilment. The second and third questions related to pharmacy interns' professional experiences that cause stress. Among the stressors were feeling inadequate/inexperienced and inadequate recognition. Factors interfering with pharmacy interns' professional expectations included time and organisational management.

The fourth and fifth questions asked about their needs to maintain professional satisfaction and fulfilment. Pharmacy interns' needs focused on organisational support and professional development. Actions that pharmacy interns identified as potentially achieving satisfaction and fulfilment included professional development and time management.

Representative examples of responses from the open-ended questions can be found in Supplementary file 3.

## 5 Discussion

This study examined resilience and empathy scores, as well as potentially associated factors, among pharmacy interns. There were no significant differences in resilience scores between the three ITP cohorts, and no predictive value of age, gender, internship settings, cultural background, previous education, or employment history was identified. These findings align with existing research,^40^^,^^41^ which similarly reported no substantial impact of these demographic variables on resilience levels among healthcare professionals and students. This suggests that resilience levels among pharmacy interns may vary, but not significantly, due to demographic or educational factors. Despite variations in the internship experiences caused by the COVID-19 pandemic, the mean resilience scores across the ITP cohorts remained consistent between the years (2020–2022). Although only a few studies allow for a comparison of CD-RISC scores of pharmacists, it seems that pharmacy interns in this study have a lower resilience score when compared to other pharmacist cohorts who have also been studied using the CD-RISC-25.^42^^,^^43^ For instance, the mean resilience score of pharmacy interns in this study of 66.6 (SD 11.7) was comparatively lower than that of community pharmacists in Lebanon during the COVID-19 pandemic,^42^ as well as final year pharmacy students in the UK in 2022.^43^ Pharmacy interns showed lower mean resilience scores than final-year nursing students in the USA in 2018,^44^ as well as a community group in Australia comprising three different age groups.^45^ These results indicate that pharmacy interns may benefit from support in developing or strengthening their resilience to manage the demands and challenges of their profession effectively.

The mean empathy score of 84.3 (SD 9.23) was relatively high compared to other studies using the KCES. For example, a study with nursing students in the US reported mean scores of 77.6 (SD 6.92) pre-intervention and 80.3 (SD 4.11) post-intervention,^46^ while a study with pharmacy students showed a mean score of 82.1 (SD 8.5) pre-intervention, which increased to 84.4 (SD 8.8) following behavioural change intervention.^47^ Simko et al. (2021) found an increase from 83.8 (SD 7.8) to 89.7 (SD 7.35) post-intervention among students in several health sciences disciplines.^48^ The seemingly high levels of empathy observed may be attributed to interns' experiential experiences and real-world encounters with patients, which facilitate a more holistic understanding of patient care and the development of empathy.^49^

Irrespective of the different internship experiences caused by the COVID-19 pandemic, there was consistency in the mean empathy scores across the ITP cohorts, similar to the resilience results. While the lack of predictive value in gender, internship settings, cultural background, or previous education seems in contradiction to common assumptions and some prior studies that suggest women in general have higher empathetic concerns^50^ and that cultural background influences empathy,^51^ it aligns with other research indicating that any associations may be more complex and context-dependent.^52^ The personal characteristics of the pharmacy interns involved, the specific constructs measured by the KCES scale, and the unique context of the Australian ITP may have influenced these findings.

This study found differences in empathy scores between age groups, which is consistent with previous research indicating that empathy tends to diminish slightly with age.^53^, ^54^, ^55^ There is a growing concern that empathy also diminishes over time for healthcare students and professionals, particularly after entering clinical practice.^56^

Interns who worked part-time pre-internship, most likely during their undergraduate degree, were found to have a significantly higher level of empathy than those who worked full-time as students. Previous work experience can increase empathy, for example, with nursing students.^57^ The difference in empathy scores between interns working part-time or full-time as students may be related to them being able to manage their workload and study responsibilities more effectively when working part-time rather than full-time, resulting in better self-care and a lower risk of burnout. In contrast, pharmacy students who work full-time are likely to struggle balancing their work and study commitments, leading to increased stress and risk of burnout and possibly depletion of empathy.^58^^,^^59^ Longer work hours are associated with decreased employee empathy levels, indicating that extended hours may compromise an individual's emotional ability to connect with others may lead to burnout, which can further reduce a person's ability to empathise.^60^ It should be noted that this study did not collect data on whether the pre-internship work occurred in a pharmacy or non-pharmacy setting.

Overall, the open-ended results of our study complement the findings of the original survey by Schommer et al. (2020),^34^ underscoring the importance of addressing common stressors and providing organisational support and opportunities for professional development to enhance the satisfaction and well-being of pharmacy interns and pharmacists.^61^ Pharmacy interns mentioning ‘helping patients’ most frequently as giving them fulfilment seems congruent with their relatively high empathy levels. Additional themes, centred on the COVID-19 pandemic, ITP requirements, and feelings of inadequacy and inexperience among pharmacy interns. The pandemic created significant challenges for the pharmacy profession, and pharmacy interns had to adapt to new protocols and safety measures to ensure the health and safety of themselves and their patients. The pandemic-related changes seemed to increase the workload and stress for pharmacy interns as they tried to complete changing ITP requirements. They may have also disrupted their professional development plans and career opportunities. Providing support mechanisms and opportunities for professional development may mitigate the unique challenges faced by pharmacy interns in the current healthcare landscape.

An association between resilience and empathy levels among pharmacy interns was not established, similarly to observations by McFarland et al. (2017) with oncology medical house officers.^62^ One explanation for the lack of association could be that resilience and empathy are distinct constructs that may be influenced by different factors. As an example, resilience may be influenced by factors such as self-regulation, coping strategies and social support, while empathy may be influenced by factors such as cognitive and affective processes, social experiences, and cultural background. Additionally, pharmacy interns, who are early in their professional career, may still be developing these skills, further complicating the relationship between these attributes. Thus, while high-resilience individuals may be better equipped to cope with stress and adversity, they may not necessarily be more empathetic towards others. However, a more recent study by Brown et al. (2022) reported a significant positive correlation between changes in resilience scores and changes in empathy scores, indicating that higher levels of resilience or strengthening resilience may lead to higher levels of empathy.^63^ Their findings support the notion that improving resilience can help prevent empathy erosion, as previously suggested by Shapiro (2011).^64^

As this cohort of pharmacy interns seemingly had lower mean resilience scores and higher mean empathy scores compared to similar populations, fostering their resilience would be a priority. Enhancing the resilience of pharmacy interns through targeted interventions can potentially improve their overall well-being and job performance. Several successful resilience programs have been offered to pharmacy interns, many based on or including mindfulness-based training.^65^, ^66^, ^67^ Synergistically, research suggests that this may also increase empathy levels.^68^^,^^69^

Fostering resilience among pharmacy professionals early in their careers will contribute to reducing burnout and attrition from the profession. By developing resilience through effective coping strategies, self-care practices, and a positive mindset, pharmacy professionals can better manage their stress and maintain their well-being. In addition to appropriate organisational support, this can improve job satisfaction, decrease burnout rates, and ultimately lead to reduced attrition.

A number of strengths and limitations apply to this study. The use of validated psychometric tools provided a reliable assessment of both resilience and empathy scores among participants. Observations of different cohorts over three years allowed for a more comprehensive understanding of the impact of the COVID-19 pandemic on internship experiences and resilience and empathy over time. By focusing specifically on pharmacy interns, the study was able to shed light on a group of healthcare professionals who are often overlooked in research studies. The study has limitations, including being conducted in a single accredited Australian internship training program with a small sample size. The use of convenience sampling from ITP workshop attendees and paper-based surveys could introduce selection bias, possibly limiting the generalisability of the findings. As both scales used in this study were self-reported measures, social desirability bias and response bias may exist. It is also important to acknowledge that this study solely focused on individual resilience and, thus, does not provide any information regarding how an organisation's work environment and culture can support employee well-being and prevent burnout.

## 6 Conclusion

This study investigated the individual levels of resilience and empathy of pharmacy interns. Findings emphasise the need for ongoing professional development opportunities and support mechanisms to enhance their well-being and job satisfaction. In addition to organisational support and workload management, targeted interventions to foster resilience may ensure their success in managing the profession's demands and challenges and facilitate the success and well-being of pharmacy interns in their profession, ultimately leading to enhanced job performance and satisfaction, reduced burnout and attrition from the profession.

## Funding

This research did not receive any specific grant from funding agencies in the public, commercial, or not-for-profit sectors.

## Declaration of Competing Interest

The authors declare that they have no known competing financial interests or personal relationships that could have appeared to influence the work reported in this paper.

## Appendix A Supplementary data

Supplementary material Image 1
