# Supplementary material for: Resilience and empathy in pharmacy interns: Insights from a three-year cohort study
Source: Explor Res Clin Soc Pharm. 2023 Sep 24;12:100333. doi: 10.1016/j.rcsop.2023.100333 (PMC10562191; doi:10.1016/j.rcsop.2023.100333)
Supplement: Supplementary file 1 — Supplementary material [file mmc1.docx]

**Supplementary file**

Supplementary file 1: CROSS Guidelines

| **Section/topic** | **Item** | **Item description** | **Reported section** |
| --- | --- | --- | --- |
| **Title and abstract** | | |  |
| Title and abstract | 1a | State the word “survey” along with a commonly used term in title or abstract to introduce the study’s design. | Abstract |
|  | 1b | Provide an informative summary in the abstract, covering background, objectives, methods, findings/results, interpretation/discussion, and conclusions. | Abstract |
| **Introduction** | | |  |
| Background | 2 | Provide a background about the rationale of study, what has been previously done, and why this survey is needed. | Introduction |
| Purpose/aim | 3 | Identify specific purposes, aims, goals, or objectives of the study. | Introduction |
| **Methods** | | |  |
| Study design | 4 | Specify the study design in the methods section with a commonly used term (e.g., cross-sectional or longitudinal). | Methods |
|  | 5a | Describe the questionnaire (e.g., number of sections, number of questions, number and names of instruments used). | Methods |
| Data collection methods | 5b | Describe all questionnaire instruments that were used in the survey to measure particular concepts. Report target population, reported validity and reliability information, scoring/classification procedure, and reference links (if any). | Methods |
|  | 5c | Provide information on pretesting of the questionnaire, if performed (in the article or in an online supplement). Report the method of pretesting, number of times questionnaire was pre-tested, number and demographics of participants used for pretesting, and the level of similarity of demographics between pre-testing participants and sample population. | Methods |
|  | 5d | Questionnaire if possible, should be fully provided (in the article, or as appendices or as an online supplement). | Supporting information and |
| Sample characteristics | 6a | Describe the study population (i.e., background, locations, eligibility criteria for participant inclusion in survey, exclusion criteria). | methods |
|  | 6b | Describe the sampling techniques used (e.g., single stage or multistage sampling, simple random sampling, stratified sampling, cluster sampling, convenience sampling). Specify the locations of sample participants whenever clustered sampling was applied. | Methods |
|  | 6c | Provide information on sample size, along with details of sample size calculation. | N/A |
|  | 6d | Describe how representative the sample is of the study population (or target population if possible), particularly for population-based surveys. | Discussions |
| Survey  administration | 7a | Provide information on modes of questionnaire administration, including the type and number of contacts, the location where the survey was conducted (e.g., outpatient room or by use of online tools, such as SurveyMonkey). | Methods |
|  | 7b | Provide information of survey’s time frame, such as periods of recruitment, exposure, and follow-up days. | Methods |
|  | 7c | Provide information on the entry process:  –>For non-web-based surveys, provide approaches to minimise human error in data entry.  –>For web-based surveys, provide approaches to prevent “multiple participation” of participants. | Methods |
| Study preparation | 8 | Describe any preparation process before conducting the survey (e.g., interviewers’ training process, advertising the survey). | Methods |
| Ethical considerations | 9a | Provide information on ethical approval for the survey if obtained, including informed consent, institutional review board [IRB] approval, Helsinki declaration, and good clinical practice [GCP] declaration (as appropriate). | Methods |
|  | 9b | Provide information about survey anonymity and confidentiality and describe what mechanisms were used to protect unauthorised access. | Methods |
| Statistical  analysis | 10a | Describe statistical methods and analytical approach. Report the statistical software that was used for data analysis. | Methods |
|  | 10b | Report any modification of variables used in the analysis, along with reference (if available). | N/A |
|  | 10c | Report details about how missing data was handled. Include rate of missing items, missing data mechanism (i.e., missing completely at random [MCAR], missing at random [MAR] or missing not at random [MNAR]) and methods used to deal with missing data (e.g., multiple imputation). | Methods |
|  | 10d | State how non-response error was addressed. | Results |
|  | 10e | For longitudinal surveys, state how loss to follow-up was addressed. | N/A |
|  | 10f | Indicate whether any methods such as weighting of items or propensity scores have been used to adjust for non-representativeness of the sample. | N/A |
|  | 10g | Describe any sensitivity analysis conducted. | N/A |
| **Results** | | |  |
| Respondent characteristics | 11a | Report numbers of individuals at each stage of the study. Consider using a flow diagram, if possible. | Results |
|  | 11b | Provide reasons for non-participation at each stage, if possible. | Results |
|  | 11c | Report response rate, present the definition of response rate or the formula used to calculate response rate. | Results |
|  | 11d | Provide information to define how unique visitors are determined. Report number of unique visitors along with relevant proportions (e.g., view proportion, participation proportion, completion proportion). | Results |
| Descriptive  results | 12 | Provide characteristics of study participants, as well as information on potential confounders and assessed outcomes. | Results  and discussion |
| Main findings | 13a | Give unadjusted estimates and, if applicable, confounder-adjusted estimates along with 95% confidence intervals and p-values. | N/A |
|  | 13b | For multivariable analysis, provide information on the model building process, model fit statistics, and model assumptions (as appropriate). | N/A |
|  | 13c | Provide details about any sensitivity analysis performed. If there are considerable amount of missing data, report sensitivity analyses comparing the results of complete cases with that of the imputed dataset (if possible). | Results |
| **Discussion** | | |  |
| Limitations | 14 | Discuss the limitations of the study, considering sources of potential biases and imprecisions, such as non-representativeness of sample, study design, important uncontrolled confounders. | Discussion |
| Interpretations | 15 | Give a cautious overall interpretation of results, based on potential biases and imprecisions and suggest areas for future research. | Discussion |
| Generalisability | 16 | Discuss the external validity of the results. | Discussion |
| **Other sections** | | |  |
| Role of funding source | 17 | State whether any funding organisation has had any roles in the survey’s design, implementation, and analysis. | N/A |
| Conflict of interest | 18 | Declare any potential conflict of interest. | Conflicts of interest |
| Acknowledgements | 19 | Provide names of organisations/persons that are acknowledged along with their contribution to the research. | Acknowledgements |

Supplementary file 2: Association of demographic characteristics with overall CD-RISC-25 and KCES scores

| Characteristics | Overall CD-RISC-25 score | Overall KCES score |
| --- | --- | --- |
| ITP Cohort | P value > 0.05^+^ | P value > 0.05^*^ |
| Gender | P value > 0.05^^^ | P value > 0.05^#^ |
| Age | P value > 0.05^+^ | **P value < 0.05^*^** |
| Internship Settings | P value > 0.05^+^ | P value > 0.05^*^ |
| Cultural Background | P value > 0.05^+^ | P value > 0.05^*^ |
| Education | P value > 0.05^+^ | P value > 0.05^*^ |
| Pre-intern Employment History | P value > 0.05^+^ | **P value < 0.001^*^** |
| Current Employment History | P value > 0.05^^^ | P value > 0.05^#^ |

Significant P-values (< 0.05) are bolded

^ independent T-Test

+ one-way ANOVA

* Kruskal-Wallis

# Mann-Whitney

Supplementary file 3: Representative responses from the open-ended questions

| Theme | Qualitative analysis |
| --- | --- |
| 1. What are the positive things in your professional life that give you satisfaction and fulfilment? | |
| Theme 1: Helping patients | Patients outcomes that are positive and receiving a simple thank you or appreciation for providing good care  Seeing patient & helping them make their everyday somewhat easier - they are often very grateful |
| Theme 2: Professional development | Opportunity for advancement in the workplace  Continued education and seeking more opportunities within the workplace  The self-development that comes with these interactions from clinical learning to being able to get a better understanding of that group  Learning new skills |
| Theme 3: Relationships with co-workers/colleagues | Relationships with other interns and staff  Enjoyment in my work and strong relationships with my colleagues |
| Theme 4: Mentoring/feedback | Positive feedback  Receiving positive and constructive feedback, in the right way |
| Theme 5: Making a difference | Being able to connect with patients and know that your intervention made a difference |
| 2. What are the things that you view as specific stressors in your professional life? | |
| Theme 1: Feeling inadequate and inexperienced | Talking to prescribers  Not having enough experience/knowledge to provide advice and needing to defer to pharmacists or resources |
| Theme 2: Inadequate recognition | Lack of appreciation by colleagues and feeling temporary or "extra" |
| Theme 3: Time management | Time pressure in my workplace is a big stressor, I often feel pressure from other pharmacists & nursing staff to work faster to get patients discharged (our hospital has bed pressure)  Time constraints to be able to provide optional patient care  Trying to balance work, intern and personal life  Study vs full-time work vs balanced lifestyle |
| Theme 4: Organisational management | Management, constant micromanagement especially as an intern, no freedom or autonomy to make your own decisions  My preceptor/manager  Negative/toxic colleagues' attitudes/view points |
| Theme 5: ITP requirements | The pressures of the workload of intern year - having to work full time, complete training frameworks, do research, prepare education & commute, eat healthily, exercise, get enough sleep, socialise and study seems like an impossible task at times. |
| Theme 6: Angry/abusive patients and customers | Aggressive customers  When I try really hard to work out a solution for a patient and they are still really rude or don't appear to appreciate that something was able to be worked out |
| Theme 7: COVID-19 pandemic | COVID19, the potential that anyone in the hospital could have it including staff and patients. The PPE makes you more tired and I drink less water because of it which makes me feel dehydrated and not so well. I'm run down and I feel like I can't stop working because I need to meet my hours, and I have to study for the exams. I don't feel ready for the exams because I have been worried about COVID19 and how it impacts me and my family, and when I come home from work, I'm so tired I can't study. We don't really have an outlet, its work, study sleep, repeat.  Intern exams and work changes due to COVID-19 (eg, exam dates and delivery format) |
| Theme 8: Finances | Poor financial reward  Fees for exams & registration |
| 3. What interferes with your ability to meet your expectations in your professional life? | |
| Theme 1: Time management | Time stressors interferes with my ability to have the kind of quality conversations I'd like to have sometimes. Intern year time stress as aforementioned, sometimes leaves me stressed where I might otherwise be able to enjoy my work as I'm always anticipating the next thing I need to get done. |
| Theme 2: Organisational management | The power of the superior which is sometimes unfair and done out of emotions and personal judgment, instead of a professional one. |
| Theme 3: Health | Being diagnosed with Bipolar Affective Disorder (BPAD)  Fatigue from everything going on- especially this time of year like I'm thinking about 100 different things at once |
| Theme 4: Striving for perfection | At this stage as an intern, my clinical experience & knowledge, I feel this limit my ability to meet my expectations of my professional's life. However, this is improving as I feel more capable now compared to when I started my internship.  ITP program that has been chopped back to the bare minimum.  Inability to complete activities (such as essential training cancelled by Covid-19). |
| Theme 5: Relationships | Need to balance social and professional relationships |
| Theme 6: Finances | Enough money  Money to further education |
| 4. What needs do you have in your professional life that would enable you to achieve satisfaction and fulfilment? | |
| Theme 1: Organisational support | A strong supportive team that encourages me to keep learning  Support from more pharmacists in the workforce |
| Theme 2: Professional development | Variety and opportunities to improve knowledge and skills |
| Theme 3: Acknowledgement and recognition | Monetary and appreciation  Receiving positive feedback and recognition when relevant |
| Theme 4: Time management | More time/availability to speak with patients 1:1 without concurrent involvements (6 other patients and the phone ringing & nurse waiting for advice) |
| Theme 5: Relationships | Work relationships  Strong friendships with colleagues  Supportive friends and families |
| Theme 6: Health | Hobbies (reading, tv, YouTube) |
| 5. What are specific actions in your professional life that you could take to allow you to achieve satisfaction and fulfilment? | |
| Theme 1: Professional development | Move to a more clinical role  Join more groups and training for professional development |
| Theme 2: Time management | Planning my day out in the morning and committing more time to things I value  Work life balance |
| Theme 3: Mindfulness/reflection | Utilise my annual leave, make sure I am caring for myself  Reflection, specifically on the achievements and successful or meaningful interventions and conversations of the day is something I need to do more of. I tend to always focus on what didn't go well as what's next. While fixing my shortfalls is important to be competent, reflecting and playing to my strengths and success is necessary to move forward.  Mindfulness and CBT |
| Theme 4: Asking for help | Ask for guidance & resources when stuck  Nerves to ask for help at a time  Reach out for help when needed and be a pleasant worker |
